# Human native kappa opioid receptor functions not predicted by recombinant receptors: Implications for drug design

**DOI:** 10.1038/srep30797

**Published:** 2016-08-05

**Authors:** John Broad, Damien Maurel, Victor W. S. Kung, Gareth A. Hicks, Michael Schemann, Michael R. Barnes, Terrence P. Kenakin, Sébastien Granier, Gareth J. Sanger

**Affiliations:** 1 Blizard Institute, Barts and The London School of Medicine and Dentistry, Queen Mary University of London, UK; 2 Institut de Génomique Fonctionnelle, Dépt de Pharmacologie Moléculaire, UMR 5203 CNRS–U 661 INSERM, Univ Montpellier I & II, 141, 34094 Montpellier, France; 3 Tioga Pharmaceuticals, 9393 Towne Centre Drive, Suite 200, San Diego, California, USA; 4 Human Biology, TU München, D-85350 Freising-Weihenstephan, Germany; 5 William Harvey Research Institute, Barts and The London School of Medicine and Dentistry, Queen Mary University of London, UK; 6 Dept of Pharmacology, University of North Carolina School of Medicine, Chapel Hill, NC, USA

## Abstract

If activation of recombinant G protein-coupled receptors in host cells (by drugs or other ligands) has predictive value, similar data must be obtained with native receptors naturally expressed in tissues. Using mouse and human recombinant κ opioid receptors transfected into a host cell, two selectively-acting compounds (ICI204448, asimadoline) equi-effectively activated both receptors, assessed by measuring two different cell signalling pathways which were equally affected without evidence of bias. In mouse intestine, naturally expressing κ receptors within its nervous system, both compounds also equi-effectively activated the receptor, inhibiting nerve-mediated muscle contraction. However, whereas ICI204448 acted similarly in human intestine, where κ receptors are again expressed within its nervous system, asimadoline was inhibitory only at very high concentrations; instead, low concentrations of asimadoline reduced the activity of ICI204448. This demonstration of species-dependence in activation of native, not recombinant κ receptors may be explained by different mouse/human receptor structures affecting receptor expression and/or interactions with intracellular signalling pathways in native environments, to reveal differences in intrinsic efficacy between receptor agonists. These results have profound implications in drug design for κ and perhaps other receptors, in terms of recombinant-to-native receptor translation, species-dependency and possibly, a need to use human, therapeutically-relevant, not surrogate tissues.

There is considerable interest in developing kappa opioid (κ) receptor agonists to reduce pain, without causing dysphoria, addiction or constipation^1^,^2^,^3^,^4^. Drugs which do not cross the blood-brain barrier, for example, retain analgesic activity with minimal dysphoria, although intestinal motility remains inhibited^2^,^5^. Such drugs may treat a diarrhoea-predominant sub-group of patients with irritable bowel syndrome (IBS), a chronic condition characterised by abdominal pain and disturbed bowel habit^6^. Pilot studies with κ receptor agonists (fedotozine^7^; asimadoline^8^,^9^,^10^) in IBS patients have improved abdominal symptoms, reduced sensitivity to colon distension (fedotozine^11^,^12^; asimadoline^13^) and provided adequate relief in a placebo-controlled trial and when given ‘as needed’ (asimadoline^14^,^15^).

New κ receptor agonists continue to be identified^1^,^2^,^3^,^4^ aided by agonist docking, site-directed mutagenesis and crystal structure analysis^16^. These include functionally-selective κ receptor agonists which induce biased receptor signalling^1^,^4^, promising drugs which favour a therapeutically-desirable outcome rather than side-effects mediated by the same receptor in different tissues^17^. For approaches which use recombinant receptors in host cells, it is essential to translate the proposed activity of a new compound by demonstrating that derived data corresponds to the functions of the receptor in its native environment and in particular, for human, therapeutically-relevant tissues. For instance, the existence of cell-specific post-translational modifications of receptor mRNA, altering the efficiency of coupling of the receptor to intracellular pathways^18^,^19^, would lead to failure of translation.

The need to use human tissues is important because variations in receptor expression, functions and pharmacology between, for example, rodents and humans, complicates data interpretation and contributes to failed translation of novel drug candidates^20^,^21^. For the κ receptor, species-dependent variations include the ability of agonists to inhibit gastrointestinal (GI) transit of a meal in guinea-pigs and mice, not in rats^22^. In rats, guinea-pigs and pigs, κ receptors are distributed to myenteric neurons within the GI tract which control motility, with little expression by submucosal neurons which largely control intestinal secretion^23^,^24^, but in mice, the opposite is described^25^. Mouse strain- and species-dependent differences in κ receptor functions within a particular tissue^26^,^27^ creates further uncertainties over which animal best reflects human functions. These include species differences in ligand-induced receptor phosphorylation, desensitisation^28^ and biased agonism (arising from variations in receptor structures linked to cell signalling pathways^27^) and also mouse strain differences in post-translational modifications of κ receptor mRNA^29^.

The present study began by evaluating the abilities of two structurally-distinct κ receptor agonists, ICI204448^30^ and asimadoline (EMD 61753)^8^,^9^, to inhibit contractions of mouse and human isolated colon evoked by electrical stimulation of intrinsic cholinergic neurons. These contractions represent a function of the main enteric excitatory motor neuron of the colon^31^ and inhibition by opioid receptor agonists reflects at least partly, their abilities to reduce diarrhoea or cause constipation^32^,^33^. ICI204448 and asimadoline are both described as full or maximally-effective agonists at the human κ receptor, with good affinity^34^,^35^,^36^ and selectivity of action over a range of other receptors and ion channels^34^,^36^,^37^,^38^,^39^. Surprisingly, however, we found marked differences in their abilities to reduce cholinergic activity in human colon, whereas both appeared as full agonists in mouse colon^40^. These data prompted a systematic re-examination of the actions of these compounds in a range of different assays using recombinant and native κ receptors. Together, the results indicate that the differences in function cannot be attributed to variations in potency, biased agonism or functional selectivity for the intracellular Gi protein and ß-arrestin signalling pathways. Instead, structural differences between the mouse and human κ receptor may affect levels of receptor expression and/or how each receptor orthologue couples to intracellular pathways or additional proteins when the receptor is expressed within its native environment, changing the pharmacology of the receptor in species-dependent and perhaps, tissue-dependent ways. These data, involving the critical use of native human tissue, have clear and profound implications in the design of κ receptor agonists as drugs.

## Results

### Functional assays using recombinant receptors expressed in host cells

The abilities of asimadoline and ICI204448 to activate the mouse and human κ receptors were assessed using two different assays of intracellular signalling, examining activation of the G protein (using the Gi alpha subunit, measured using BRET or Bioluminescence resonance energy transfer) and internalisation of the receptor into the cell (measured as arrestin recruitment, using the BRET assay and κ receptors fused with a YFP at C-terminus).

**Figure 1:**
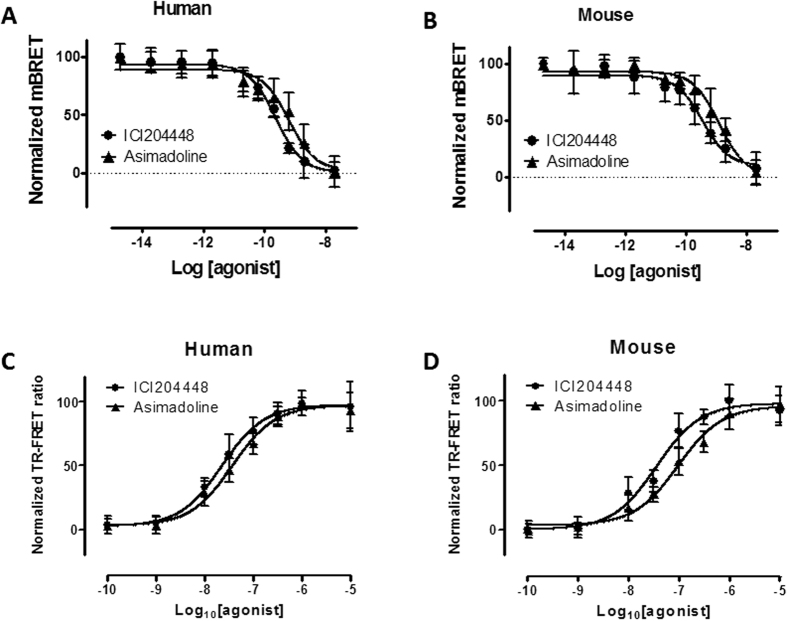
Gi activation and internalisation of κ receptors by asimadoline and ICI204448 in HEK293 cells expressing human (panels A and C) and mouse (panels B and D) κ receptors. Gi activation (panels A and B) was measured as Gi heterotrimer complex dissociation/conformational changes using bioluminescence resonance energy transfer (BRET), calculated by the ratio of emission of Venus (535 nm) to Rluc8 (480 nm) (mBRET). Receptor internalization (panels C and D) was monitored by Time-Resolved Fluorescence Energy Transfer (TR-FRET) at 37 °C, calculated as the ratio of emission of terbium cryptate (620 nm) to fluorescein (520 nm). Concentration-response curves for asimadoline and ICI204448 were obtained by measuring at steady state (80 min). For each receptor, three independent experiments were conducted for asimadoline and ICI204448, and the effects calculated as % inhibition of mBRET (Gi activation) measured at the lowest concentrations tested (normalised as 100%) and as % increase in TR-FRET ratio (internalisation) and the results expressed as means ± SD.

For both the mouse and human κ receptor, asimadoline and ICI204448 each concentration-dependently reduced BRET signalling with similar EC_50_ values (the concentration which evoked a half-maximal response; respectively 0.8 and 0.3 nM for asimadoline and 0.3 and 0.5 nM for ICI204448) and maximum activity (Fig. 1A,B; Table 1). In receptor internalisation experiments the effects of asimadoline and ICI204448 were first evaluated to determine the internalisation kinetic; all measurements were subsequently made at steady state (after 75 min) for dose-responses experiments. Under these conditions, asimadoline and ICI204448 each concentration-dependently induced receptor internalisation of both the mouse and human receptors, at similar concentrations; the EC_50_ values were respectively 123 and 55 nM for asimadoline and 50 and 33 nM for ICI204448 (Fig. 1C,D; Table 2).

### Quantification of potential signalling bias

Analysis of the affinity and efficacy of asimadoline and ICI204448 in the Gi activation and arrestin recruitment assays for both species showed only minimal evidence of biased activity of asimadoline (compared with ICI204448, asimadoline was 1.5 times biased toward producing selective Gi activation over receptor internalization; Table 1).

### Inhibition of cholinergic activity in mouse colon

**Figure 2:**
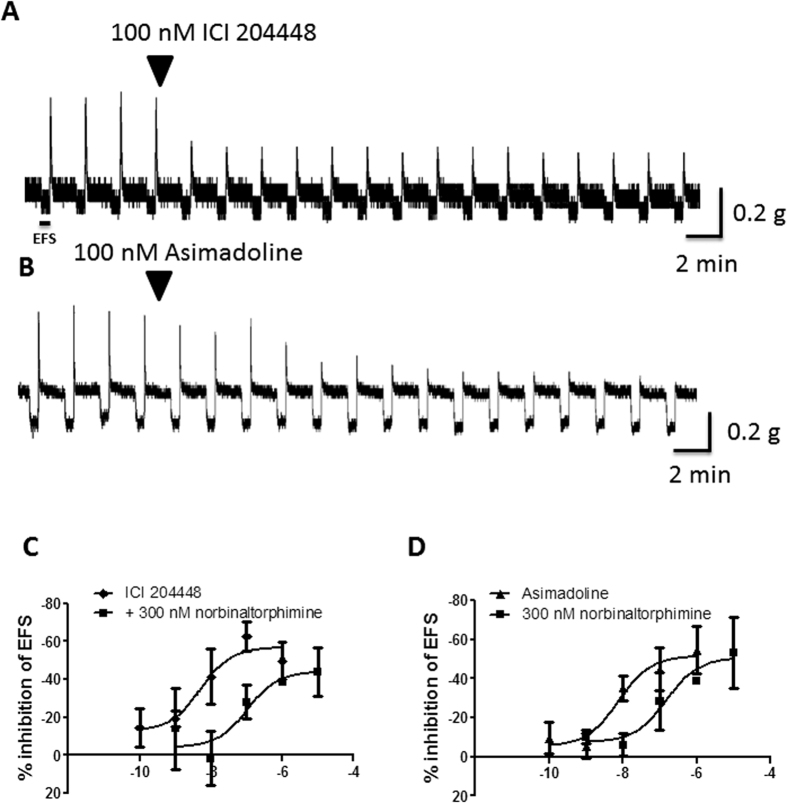
Concentration-dependent inhibition of cholinergically-mediated, EFS-evoked contractions of mouse isolated colon muscle loops. Panels A and B show representative trace examples of ICI 204448 (**A**) and asimadoline (**B**) in the proximal colon. Panels C and D show the effect of pre-incubation of the kappa opioid receptor antagonist norbinaltorphimine 300 nM on the concentration response relationship for the inhibition of EFS evoked after contractions of ICI 204448 (**C**) and asimadoline (**D**). Data are expressed as means ± SEM; n = 3–4 each concentration.

In mouse proximal and distal colon, electrical field stimulation (EFS) of the intrinsic neurons evoked a small muscle relaxation or sometimes, just inhibition of spontaneous contractions and on termination of EFS, a consistent, large-amplitude ‘after-contraction’ (Fig. 2). These responses were prevented by the neurotoxin tetrodotoxin 1 μM (n = 9 and 4, respectively). The presence of the muscarinic receptor antagonist atropine 1 μM, prevented cholinergic activity and revealed or increased EFS-evoked relaxations (e.g. by 61 ± 13% in proximal colon; n = 7) and greatly reduced after-contractions (e.g. by 81 ± 5% in proximal colon; n = 7). Relaxations were abolished by the nitric oxide synthase inhibitor L-NAME 300 μM (n = 4, 3; proximal and distal colon), indicating the involvement of nitric oxide from nitrergic neurons and confirming previous observations^41^. The effects of the κ receptor agonists were then assessed for their ability to modulate the larger, more consistent EFS-evoked, cholinergically-mediated after-contractions.

ICI204448 and asimadoline were approximately equi-effective and equi-potent inhibitors of the response to EFS with no statistically significant difference between the concentration-response curves for either compound (Fig. 2; Table 3). The E_max_ (concentration which evoked a maximal response) and EC_50_ (concentration which evoked a half-maximal response) values were determined. In proximal colon the E_max_ and *p*EC_50_ values were, respectively, 49 ± 8% and 8.5 ± 0.8 for ICI204448 and 51 ± 8% and 8.1 ± 0.5 for asimadoline. Asimadoline had a slower onset of action; the time taken for asimadoline 1 μM to achieve maximum activity was 17 ± 3 min (n = 3) whereas for ICI204448 1 μM the time to max was 7 ± 2 min (n = 4; P < 0.05). Critically, however, the presence of the selective κ receptor antagonist nor-binaltorphamine 300 nM^42^,^43^ antagonised the effects of both ICI204448 and asimadoline in mouse proximal colon in a surmountable manner, reducing the calculated *p*EC_50_ values (Fig. 2; Table 3) and confirming that in this assay, ICI204448 and asimadoline acted as κ receptor agonists. Both compounds also reduced contractions of the distal colon in a similar manner (e.g. asimadoline 1 μM and ICI204448 1 μM reduced contractions by, respectively, 59 ± 13% (n = 3) and 70 ± 5% (n = 4). Asimadoline 1 μM and ICI204448 1 μM had no consistent effects on contractions of proximal colon induced by carbachol 1 μM (respectively: 5 ± 7% (n = 4) and 0 ± 5% (n = 3) initial carbachol response).

### Inhibition of cholinergic activity in human colon

Table 4 provides details of the patients from whom colon was obtained. As previously reported^44^, the responses evoked by EFS were prevented by tetrodotoxin 1 μM and were often biphasic. During EFS an initial contraction was observed in most strips of ascending (189/280 strips studied) and descending colon (351/462), with the remainder displaying relaxation. The contractions were prevented by atropine 1 μM (n = 3 ascending, 6 descending) and when relaxations were observed, these were prevented by L-NAME 300 μM (in 63 strips from 24 ascending colon specimens and 81 strips from 28 descending colons). These tissues now contracted during EFS; in 3 further experiments the contractions were abolished by atropine 1 μM. After termination of EFS 59% of all strips from ascending colon (observed in 30/32 patients) contracted (the ‘after-contraction’). Similarly, after-contractions were observed in 92% of strips from descending colon (43/43 patients). These contractions have previously been shown to be attenuated by atropine 1 μM (56 ± 12%, n = 3) but further reduced by application of antagonists at NK_1_, NK_2_ and NK_3_ receptors (34 ± 19%, data from^44^), indicating a small involvement of tachykinergic neurons in the overall response to EFS; a residual non-cholinergic non-tachykinergic component was observed on occasion (data not shown). In the following experiments with κ receptor agonists the effects were analysed by measuring changes in amplitude of contractions during EFS since these contractions were regular in amplitude during repeated stimulation, cholinergically-mediated (completely blocked by atropine) and were inhibited by the κ receptor agonists in a more consistent manner than the after-contractions (data not shown).

**Figure 3:**
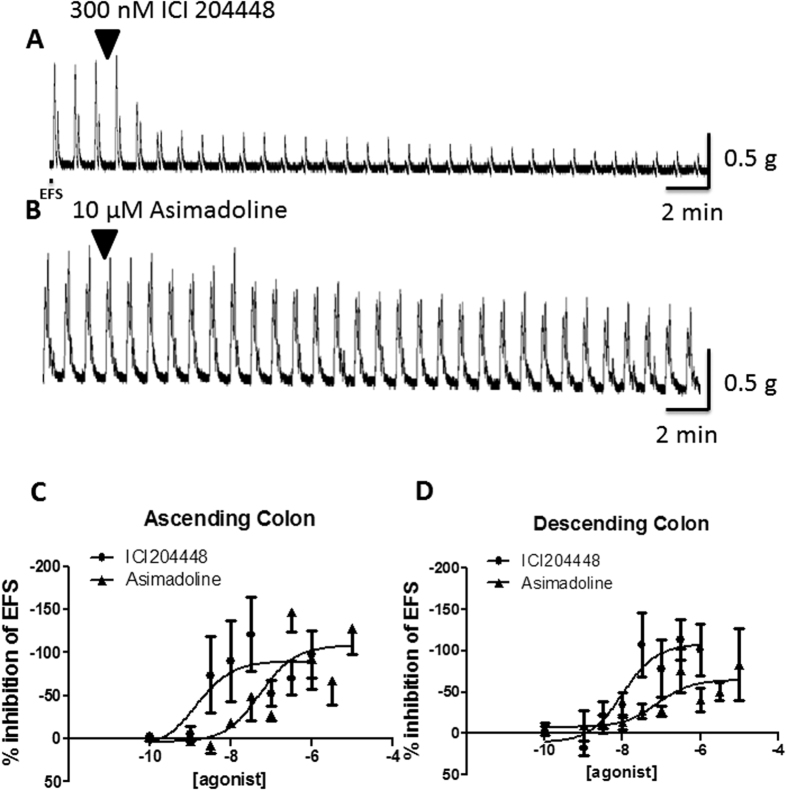
Concentration-dependent inhibition of EFS-evoked contractions of human isolated colon. Panels A and B show representative trace examples of ICI 204448 (**A**) and asimadoline (**B**). Note the slow onset of activity of asimadoline. Panels C,D show concentration response relationships for the inhibition of contractions during EFS by ICI204448 and asimadoline in ascending (**C**) and descending (**D**) human colon muscle strips. Data are expressed as means ± SEM; n = 4–5 each concentration.

ICI204448 and asimadoline each inhibited the EFS-evoked contractions but overall, there was a statistically significant difference between the concentration-response curves for these compounds (Fig. 3; Table 5). In ascending colon, this action of asimadoline appeared slowly and at concentrations higher than those for ICI204448. For example, the E_max_ and *p*EC_50_ values for ICI204448 were, respectively, 90 ± 15% and 8.9 ± 0.6 and the time to maximum inhibition at 1 μM was 7 ± 1 min. For asimadoline, the E_max_ and *p*EC_50_ values were, respectively, 108 ± 14% and 7.3 ± 0.4 and the time to maximum inhibition at 10 μM was 30 ± 5 min (n = 5, P = 0.003). Similar activity was observed in descending colon (Fig. 3; Table 5).

In further experiments using descending colon, the effect of ICI204448 was unchanged by the presence of the adrenoceptor antagonists phentolamine 1 μM and propranolol 1 μM (ICI204448 100 nM inhibited EFS-evoked contractions by 130 ± 29% EFS; n = 4), which alone had no effect on responses to EFS (2 ± 3% change from baseline EFS, n = 4); these data indicate an absence of involvement of noradrenaline in the inhibitory activity of the κ receptor agonists. During the presence of atropine 1 μM, ICI204448 100 nM had no effect on relaxations evoked by EFS (1 ± 7% change from baseline EFS; n = 3). Neither asimadoline nor ICI204448 changed baseline muscle tension in either region of colon (data not shown).

**Figure 4:**
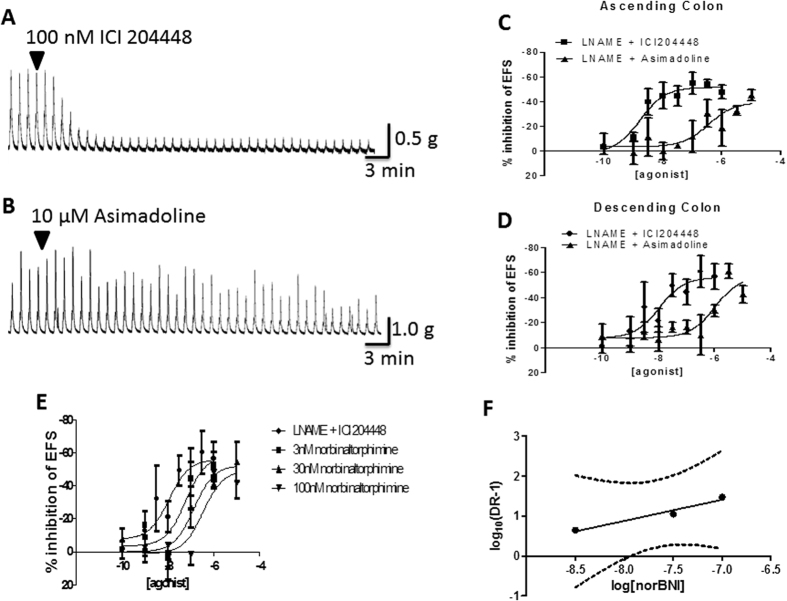
Inhibition by ICI204448 and asimadoline of cholinergically-mediated, EFS-evoked contractions of human isolated colon in the presence of L-NAME 300 μM. Representative trace examples are shown for ICI 204448 (panel A) and asimadoline (panel B). The concentration-response relationships for the inhibition of contractions by ICI204448 and asimadoline are shown in panel C for the ascending and in panel D for descending colon. Panel E shows the effect of pre-incubation of the kappa opioid receptor antagonist norbinaltorphimine 3–300 nM on the concentration-response relationship for the inhibition of contractions by ICI 2044448 in the descending colon. Panel F shows a linear regression of the relationship between log_10_[norbinaltorphimine] and the dose ratio −1 (DR-1) of the EC_50_s of ICI 204448 in the presence of 3–300 nM norbinaltorphimine. Data are expressed as means ± SEM; n = 4–6 each concentration or 3–4 each concentration in the experiments with norbinaltorphimine.

Since nitrergic and cholinergic neurons are stimulated simultaneously by EFS, each influencing the magnitude of response to the other (see above and^44^), the experiments were repeated in the presence of L-NAME 300 μM. In these conditions the ability of ICI204448 to inhibit the larger contractions of colon muscle strips was smaller than in the absence of L-NAME but the potency was unchanged and the overall difference between the concentration-response curve for ICI204448 and asimadoline remained statistically significant (Fig. 4, Table 5). The effects of asimadoline were again slower in onset and inhibitory activity was achieved only at concentrations >250 times higher than the effective concentrations of ICI204448 (Fig. 4, Table 5). In descending colon ICI204448 similarly inhibited the contractions, whereas the effects of asimadoline were achieved at concentrations 100 times greater than the effective concentrations of ICI204448 (Fig. 4, Table 5). In this region of colon, the inhibitory effects of ICI204448 were concentration-dependently antagonised by nor-binaltorphamine 3, 30 and 300 nM (n = 4 each concentration of ICI204448; slope of Schild analysis = 0.53 ± 0.11, intercept = −9.7; Fig. 4), confirming activation of κ receptors by ICI204448.

In separate experiments in which EFS was not applied, ICI204448 100 nM or asimadoline 1 μM did not affect contractions of descending colon evoked by the submaximally-effective concentration (1 μM) of carbachol (respectively, −9 ± 2%, n = 4; and −10 ± 9%, n = 4).

### Receptor occupancy studies in human colon

**Figure 5:**
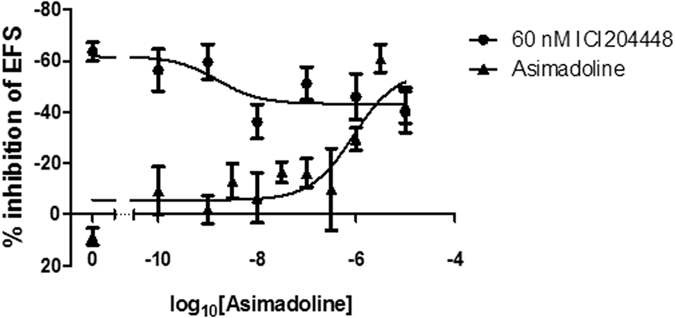
Receptor occupancy studies in human descending colon. In these experiments, conducted in the presence of L-NAME 300 μM, asimadoline or vehicle was added 30 min before application of an approximately EC_80_ concentration of ICI204448 (60 nM). The inhibition of EFS-evoked contractions caused by asimadoline alone (▲), before application of ICI204448, is shown alongside the inhibition of contractions caused by ICI204448 in the presence of asimadoline (⦁). Data are expressed as means ± SEM; n = 3–4, each concentration tested.

For clarity, these experiments were conducted using descending colon in the presence of L-NAME 300 μM. Asimadoline 100 pM–10 μM or vehicle (0.1% DMSO) was added to the bathing solution 30 min before application of an approximately EC_80_ concentration of ICI204448 (60 nM). At low concentrations of asimadoline which did not by themselves affect cholinergically-mediated contractions, and also at the higher concentrations which reduced these contractions, the ability of ICI204448 to inhibit EFS-evoked contractions was reduced. The maximum inhibition of this response to ICI204448, at concentrations of asimadoline which had no effect on the EFS-evoked contractions, was 19 ± 7%. From this, a *p*IC_50_ of 8.8 ± 0.8 was determined (n = 3–4; Fig. 5).

### Opioid receptor gene, kappa (OPRK) alignment and tertiary structural analysis

**Figure 6:**
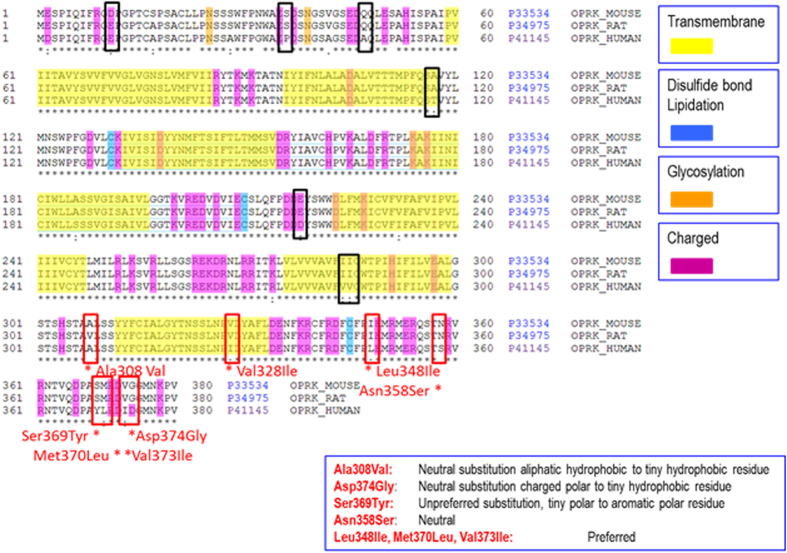
Alignment of OPRK orthologues. Human, rat and mouse OPRK orthologues were aligned using ClustalX. Amino acid alterations in the third intracellular loop and C-terminus of OPRK were evaluated for potential impact on G-protein coupling, which is known to occur particularly at intracellular charged residues (indicated in magenta) in the 3rd Intracellular loop and C-terminal region. In the 3rd Intracellular loop region, Ala308Val is a neutral substitution in rat from an aliphatic hydrophobic residue to a tiny hydrophobic residue. In the C-terminal region in rat and mouse, Leu348Ile and Ser358Asn are preferred and neutral substitutions respectively, however both are immediately adjacent to charged residues, therefore impact on G-protein coupling cannot be excluded. Four residues across a 6 amino acid region would appear most likely to impact G-protein coupling. The residues which include Tyr369Ser (unpreferred substitution of aromatic polar to tiny polar residue), Leu370Met (preferred), Ile373Val (preferred), Asp374Gly (neutral substitution of charged polar residue to a tiny hydrophobic residue).

Human, rat and mouse OPRK orthologues were aligned using ClustalX (Fig. 6). Amino acid alterations in the third intracellular loop and C-terminus of OPRK were evaluated for potential impact on G-protein coupling, which is known to occur particularly at intracellular charged residues (indicated in magenta) in the 3rd Intracellular loop and C-terminal region^45^. In the 3rd Intracellular loop region, Ala308Val is a neutral substitution in rat from an aliphatic hydrophobic residue to a tiny hydrophobic residue. In the C-terminal region in rat and mouse, Leu348Ile and Ser358Asn are preferred and neutral substitutions respectively, however both are immediately adjacent to charged residues, therefore impact on G-protein coupling cannot be excluded. Four residues across a 6 amino acid region appear most likely to impact G-protein coupling. These are Tyr369Ser (unpreferred substitution of aromatic polar to tiny polar residue), Leu370Met (preferred), Ile373Val (preferred) and Asp374Gly (neutral substitution of charged polar residue to a tiny hydrophobic residue). Several of the rodent-specific residues in OPRK have been evaluated in previous studies and are known to mediate aspects of receptor function ( www.phosphosite.org/proteinAction.do?id=3388). McLaughlin *et al.*^46^ demonstrated that GRK-mediated phosphorylation of serine 369 mediates rat OPRK desensitization and internalization. Schattauer *et al.*^27^ also showed that Ser-369 phosphorylation site in rat OPRK is required for G protein receptor kinase/arrestin-dependent p38 activation, but Ser-358 activates p38 MAPK through a phosphorylation and arrestin-dependent mechanism. Human and rodent differences at both residues lead to differing p38 activation for some OPRK ligands. Combining all of these observations in an examination of a tertiary structure model of OPRK (Supplementary Data 1) demonstrates observable differences in the stearic properties of the amino acid residues altered between human and rodents, providing further support for functional alteration.

## Discussion

The present experiments, using the κ receptor agonists ICI204448 and asimadoline, demonstrate a marked ligand- and species-dependent ability to inhibit cholinergically-mediated contractions in mouse and human colon. This was surprising as both compounds are reported as full agonists, with good affinity (Ki values of 2.69 and 0.17 or 0.6 nM) and selectivity of activity (relative to μ and δ receptors and voltage-activated sodium channels) at the κ receptor (see Introduction)^47^. Further, the differences in efficacy could not be related to differences in rate of onset of activity (slow for asimadoline, relative to ICI204448), since for each compound this was similar in both species; the reason for the differences in onset of activity is unknown although one explanation might be a difference in the rate at which each compound diffuses into the tissue. Regardless, additional experiments were initiated to understand the ligand- and species-dependent differences in efficacy.

In the proximal and distal mouse colon ICI204448 and asimadoline behaved similarly, inhibiting cholinergic activity by acting at the κ receptor (responses in proximal colon were antagonised by the selective κ receptor antagonist nor-binaltorphamine, at a concentration consistent with the range of concentrations reported to antagonise at this receptor^48^), probably by reducing ACh release from intrinsic cholinergic motor neurons (contractions evoked by a submaximally-effective concentration of carbachol were unaffected). The efficacy and nM potency of both compounds were similar to each other in both regions of colon, and similar *p*EC_50_ values were calculated from the data. Within the limits naturally imposed by the use of very different assay techniques, these *p*EC_50_ values were also broadly consistent with the nM concentrations reported as EC_50_ values in the present experiments measuring Gi activation in HEK293 cells expressing the mouse receptor (respectively 0.3 and 0.8 nM for ICI204448 and asimadoline) and with experiments by others using radioligand binding assays (IC_50_’s of 5.6 and 13 nM, respectively, in guinea-pig cerebellum and 6.6 nM for ICI204448 using mouse κ receptors in COS-1 cells^8^,^9^,^49^) and functional studies with different animal tissues (EC_50_’s of 54.5 and 15 nM, respectively, in rabbit vas deferens where both compounds behaved as full agonists^8^,^9^). Small differences in nM concentrations between each of these assays most likely reflect experimental conditions, such as the need for ligands to access receptors in cells or intact tissues, or act against different backgrounds of cell/tissue activity. These data are also consistent with previous observations in which different κ receptor agonists were found to inhibit cholinergic neuromuscular transmission and motility in various animal isolated intestine preparations, indicative of their abilities to reduce intestinal motility *in vivo* (guinea-pig^22^,^50^,^51^; rat^53^; dog^7^). Interestingly, in the mouse colon nor-binaltorphamine did not by itself change the response to EFS, indicating a lack of activity of endogenous opioids at the κ receptor; these data contrast with reported actions of κ receptor antagonists in similar studies using guinea-pig colon, suggesting potential species-dependency in how endogenous opioids affect bowel functions^33^,^51^,^54^.

In experiments measuring Gi activation in HEK293 cells expressing the human κ receptor, ICI204448 and asimadoline were approximately equi-potent and appeared to act as full agonists, relative to each other. However, whereas in human ascending and descending colon low concentrations of ICI204448 inhibited cholinergically-mediated contractions by acting at the κ receptor (the effects were prevented by nor-binaltorphamine, which by itself did not affect contractions to EFS) to reduce ACh release (contractions evoked by carbachol were unaffected by ICI204448), asimadoline was effective only at high, μM concentrations. For ICI204448, the effective concentrations were broadly similar to the range of nM concentrations which were effective in the human and mouse receptor/Gi assays, in the mouse colon (see above) and in various studies reported by others using human κ receptor binding or cell-based functional assays (respectively, Ki 2.69 nM and EC_50_’s of 19 and 4.22 nM^4^,^34^,^35^); again, small differences in values are likely to reflect differences in assay conditions. These data with ICI204448 are also broadly consistent with two similar studies using human colon and different κ receptor agonists^55^,^56^. In one other study^57^ the κ receptor agonist fedotozine inconsistently reduced ACh released by electrical stimulation of human colon but consistently reduced contractions evoked by ACh. However, the marginal selectivity of fedotozine for the κ receptor (∼2-fold over other opioid receptors in dog intestine^7^) and additional actions unrelated to the κ receptor^58^ may explain these anomalous data.

Asimadoline inhibited cholinergically-mediated contractions of human colon only at high concentrations (e.g in ascending colon, *p*EC_50_ = 7.9), far in excess of those needed to act at the κ receptor in cell-based assays (e.g. EC_50_ of 0.3 nM in the present experiments with the human receptor measuring Gi activation, and Ki’s of 0.17 and 0.6 nM in other radioligand binding studies^35^,^36^) or in the present experiments with mouse colon. Indeed, this small inhibition by high concentrations of asimadoline was not always consistent, making it difficult to confirm that it was not due to κ receptor activation by attempting to antagonise with nor-binaltorphamine. Nevertheless, it is known that at these high concentrations asimadoline can block tetrodotoxin-sensitive Na^+^ channels (EC_50_ 8.4 μM in rats^39^), activate μ-opioid receptors (3 μM in rats^8^) and block K^+^ channels in mouse epithelia (IC_50_ 23.7 μM^59^), each of which could contribute to the observed ability of asimadoline to inhibit cholinergically-mediated contractions of human colon. These data also suggest a low propensity of asimadoline to cause constipation in humans, relative to ICI204448. Notably, asimadoline is reported not to affect colonic transit or postprandial motility in normal healthy volunteers (at doses which reduce sensation to colonic distension^60^) and although diarrhoea was reduced in diarrhoea-predominant IBS patients this did not lead to constipation^14^. Significantly, however, in different experimental studies asimadoline reduced the sensation or pain caused by colonic distension^12^,^13^,^60^, suggesting that the inability of asimadoline to reduce cholinergic activity in human colon or cause constipation in humans is not due to an inability to act as an agonist at the human κ receptor in its native environment when expressed by a different type of cell.

Several reasons could explain why asimadoline failed to act as a κ receptor agonist in human colon yet behaved as an efficacious agonist in mouse colon. Schattauer *et al.*^27^ found that C-terminal amino acids most likely involved in coupling to intracellular signalling pathways are not conserved between rat and human κ receptors, arguing that these differences explain rat-human differences in how compounds might internalise the receptor. A similar comparison was therefore made between the mouse and human κ receptors. Similar differences were noted including several charge neutralising amino acid substitutions which would be expected to alter the efficacy of intracellular signalling, for example through G-proteins. These observations are consistent with the previous rat studies, indicating commonality in κ receptor structures and functions among these two examples of the rodent order, and also providing a possible explanation for the functional data obtained in the present study.

There is potential for G protein-biased μ opioid receptor agonists to exert analgesic activity in mice but with reduced ability to cause constipation^61^. The latter are thought to be more closely associated with ß-arrestin signalling and μ receptor internalization^62^,^63^,^64^; ß-arrestin-2 has also been shown to co-localise with choline acetyltransferase, calretinin and μ opioid receptor expression in the myenteric plexus of mouse intestine^65^. Similarly, δ opioid receptor functions may be changed by agonist-specific recruitment of different arrestin isoforms^66^. Finally, it has been hypothesised that κ receptor agonists which poorly activate the arrestin pathway retain analgesic activity but have reduced potential to cause dysphoria^4^. Together, these observations suggest that different functions of the opioid receptors could be evoked by biased receptor signalling, arguably contributing to significant differences in efficacy profiles between commonly-used μ opioid receptor agonist drugs^67^. However, links between the κ receptor, arrestin signalling and the potential to cause constipation have not previously been investigated. For this reason, the effects of ICI204448 and asimadoline on Gi and internalisation pathways (which reflect the level of arrestin recruitment) were examined in HEK293 cells transfected with mouse and human κ receptors. Notably, both compounds behaved with approximately similar potency and efficacy. Calculations to identify potential bias in how these compounds might activate the Gi and internalisation pathways revealed only a small (1.5-fold) bias of asimadoline towards Gi activation in both species, relative to the effects of ICI204448. This small difference is unlikely to explain the large difference in potency observed between the two compounds in human isolated colon. ICI204448 has previously been shown to have only weak propensity for biased activity towards activating the human κ receptor arrestin pathway^4^.

As it was unclear as to whether asimadoline was acting as a partial agonist at the kappa opioid receptor in human colon, receptor occupancy studies were performed to look for an ability of asimadoline to antagonise the inhibitory activity of ICI204448 in human colon. The results, showing that 30 min pre-incubation with low concentrations of asimadoline could reduce the effects of ICI204448, suggest a number of possibilities.

1. When compared with ICI204448, asimadoline may have lower intrinsic activity at the recombinant human κ receptor but this might be observed only when the receptor is expressed at low levels in cell-based assays. Further experiments are needed to test this idea.
2. In contrast with the mouse colon, enteric cholinergic neurons within human colon may not express spare receptors. This would mean that asimadoline could only act as a partial agonist in human colon but the existence of spare receptors in the mouse colon would allow this compound to exert greater efficacy (inhibiting cholinergic activity) and appear as a full agonist. The use of an irreversible κ receptor antagonist in experiments using mouse colon would test this hypothesis. Thus if spare receptors were present, the antagonist should cause surmountable antagonism prior to suppression of maximum activity at higher concentrations.
3. Another possibility is that when the κ receptor is expressed naturally in human enteric cholinergic neurons it may be poorly coupled to signalling pathways, relative to the receptor expressed in enteric cholinergic neurons of the mouse. Thus, differences between full and partial agonists would become apparent. This could be caused by variations in charged amino acids at the C-terminus of the mouse and human receptor, perhaps exacerbated by post-translational modifications of κ receptor mRNA within different tissues, changing levels of receptor coupling and/or expression in a cell-dependent manner and hence, ligand receptor efficacy^18^,^19^.
4. Asimadoline could still behave as a full agonist in human cells or tissues in which the functions of the κ receptor are not limited by an absence of spare receptors or by poor receptor coupling. This would include cells outside the gastrointestinal tract such as those mediating the previously-demonstrated analgesic activity of asimadolone^13^.
5. Although the existence of κ receptor subtypes seems unlikely, given that only one κ receptor cDNA clone has been reported and no receptor variants have been identified, it is possible that in the native environment the κ receptor could interact with another receptor or protein, changing its pharmacology in a species-dependent manner^46^. This has been demonstrated for the cloned κ receptor which can functionally couple to N-type calcium channels^68^; such couplings may alter the ability of the receptor to recognise the ligand or change the ability of the receptor to communicate with intracellular signalling pathways, possibly in a biased manner and hence, affect its efficacy^17^.

Thus in summary, the present experiments demonstrate a marked ligand- and species-dependent difference in the efficacy of at least two different κ receptor agonists in human and mouse isolated colon, most likely explained by differences in intrinsic activities of the compounds at the receptor revealed by differences in how the receptors in their native environments are expressed or coupled to intracellular pathways or other proteins. The extent to which different κ receptor agonists replicate the actions of either asimadoline or ICI204448 is unknown (in a pilot study with three different κ receptor agonists, characterised by others in cell-based assays, we demonstrated approximately similar efficacy as ICI204448 in human isolated intestine; Supplementary Data 2), being dependent on the chemical template used and the degree of subsequent structural modification. As such, these data have clear and important implications in new drug design for G protein-coupled receptor agonists since they imply that false information may be derived by reliance on assays measuring receptor functions in host cells, in animal tissues and possibly, in tissues which have little or no human therapeutic importance.

## Methods

### Functional assays using recombinant receptors expressed in host cells

Using HEK293 cells expressing human or mouse κ receptors, concentration-response curves were established in assays for Gi activation (measuring Gi heterotrimer complex dissociation/conformational changes using bioluminescence resonance energy transfer; BRET) and arrestin recruitment (measuring receptor internalization; BRET assay; κ receptors fused with a YFP at C-terminus). In all experiments, the cells were grown in DMEM supplemented with 10% FBS at 37 °C, 5% CO_2_.

For measurement of Gi activation, HEK293 cells were seeded into 96-well white plates at a density of 100,000 cells per well. The cells were transfected with 4 plasmids encoding the human κ receptor, the Gαi1 protein fused to *Renilla Luciferase8* (Gαi1-Rluc8: donor) and the β2 and Venus-γ2 G protein subunits (provided by the ARPEGE platform), at a 1:1:1:1 ratio. Cells were transfected directly into the plate using lipofectamine 2000 reagents as described by the manufacturer (Invitrogen). 24 h after transfection, cells were washed twice with PBS. Basal conditions were achieved by the addition of PBS 40 μl, followed by 10 μl coelenterazine H (5 μM total). To evaluate the effects of κ receptor ligands, the addition of PBS 30 μl was followed by addition of 10 μl coelenterazine H (5 μM total) and then 10 μl of each concentration of ligand. Activation of the κ receptors promotes dissociation of the Gαβγ protein complex resulting in the BRET signal decay. BRET between Rluc8 and Venus was measured after addition of the Rluc substrate coelenterazine H (5 μM). BRET readings were collected using a Mithras 2 plate reader. The BRET signal was calculated by the ratio of emission of Venus (535 nm) to Rluc8 (480 nm): mBRET = {(Ratio 535/480)assay − (Ratio 535/480)Rluc8 alone)} X 1000.

Receptor internalization was monitored by TR-FRET (Time-Resolved FRET). HEK293 cells were transfected with one plasmid encoding the SNAP-tagged κ receptor. 24 h after transfection, cells were labelled with 100 nM BG-Tb (benzylguanine-Terbium cryptate) for 1 h at 37 °C in DMEM supplemented with 10% FBS. After four washing steps with PBS, the fluorescence signal from BG-Tb was measured on an INFINITE F500 (TECAN) plate reader with an excitation at 337 nm and emission at 620 nm. After adding 24 μM fluorescein with increasing concentrations of κ receptor ligands, the internalization process was followed at 37 °C during 70–80 min. The signal was calculated by the ratio of emission of terbium cryptate (620 nm) to fluorescein (520 nm): ΔR = (Ratio 620/520) X 10,000.

The procedures for activating the G protein Gi (alpha subunit) were validated by control experiments. Firstly, for each experiment mouse and human κ receptor expression on the cell membrane was confirmed (by SNAP-tag labelling with BG-Tb); overall, the levels of expression were similar for both receptors (Supplementary Data 3). Secondly, non-specific activity of the κ receptor ligands on the BRET (bioluminescence resonance energy transfer) signal was determined using human embryonic kidney cells, type 293 (HEK293), transfected with the Gαi1 protein complex alone (no receptor) and exposed to increasing concentrations of ICI204448 and asimadoline (Supplementary Data 4). Finally, in each experiment, a negative and positive control was added. For the negative control, the vasopressin V_2_ receptor coupled to Gs was co-transfected with the Gαi1 protein complex and stimulated with Arginine-Vasopressin (AVP) (Supplementary Data 5). As a positive control, the μ-opioid receptor coupled to Gi was co-transfected with the Gαi1 protein complex and stimulated with DALDA (H-Tyr-D-Arg-Phe-Lys-NH_2_; Supplementary Data 5). Similarly, in each internalisation experiment with the mouse and human receptor, the level of SNAP-KOR expression was confirmed and shown to not significantly differ between the two receptors. For each experiment the vasopressin V_2_ receptor was used as a positive control (Supplementary Data 6).

### Quantification of potential signalling bia

The concentration-response curves derived from the Gi activation and arrestin assays were fitted to a mathematical model based on the Black and Leff 69 operational model to generate log(τ/K_A_) values^17^. In brief, bias is defined as ΔLog(τ/K_A_) or ΔLog(max/EC_50_) assuming n = 1 and where 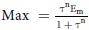 and 
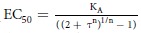
. This value represents the affinity and efficacy of the ligand for G protein activation or arrestin mobilization. The model incorporates receptor density and coupling within a system and is independent of receptor expression.

### Inhibition of cholinergic activity in mouse colon

Adult male C57BLK6/J mice (25–30 g) were culled by cervical dislocation followed by confirmation of death by cessation of blood circulation, in accordance with the UK Animals (Scientific Procedures) Act 1986. The protocol was approved by the AWERB (Animal welfare ethical review board) for Queen Mary University of London and those involved were trained and signed off as competent by the Named Training and Competency Officer and Named Animal Care and Welfare Officer. All efforts were made to minimise the number of mice used. The proximal and distal regions of colon were blunt-dissected and placed in Krebs solution (mmolL^−1^: NaCl 121.5, CaCl_2_ 2.5, KH_2_PO_4_ 1.2, KCl 4.7, MgSO_4_ 1.2, NaHCO_3_ 25, glucose 5.6) equilibrated with 5% CO_2_ in O_2_ at room temperature.

Mucosa-intact loops, cut parallel to the circular muscle of the colon were mounted between two platinum ring electrodes in 5 ml tissue baths containing Krebs solution at 37 °C and gassed with 5% CO_2_ in O_2_. Changes in muscle tension were recorded using isometric force transducers (AD Instruments, Chalgrove, UK) on a data acquisition system (Biopac Systems Inc., Goleta, CA, USA). The strips were given 1 g tension and allowed to recover for 30 min during which time the bath solutions were changed every 15 min; this initial tension was previously determined as the optimum required to maximally detect EFS-evoked responses in both regions (data not shown). Electrical Field Stimulation (EFS) was applied at 5 Hz using a maximally-effective voltage (80 V) and 0.5 ms bipolar pulse duration pulse duration, given for 30 s every 120 s, for at least 1 hour before drug addition. These parameters of EFS consistently evoked nerve-mediated responses with a good signal-to-noise ratio over spontaneous muscle activity. After obtaining consistent responses, drugs were applied non-cumulatively to separate tissues (20 min contact); changes in resting muscle tension and responses to EFS were measured. Finally, any effects of the drugs on carbachol-induced contractions were examined by first obtaining consistent responses to a submaximally-effective concentration of carbachol (1 μM; 27 ± 9% max, 5 min contact; n = 4). The compounds were then added 5 min before application of carbachol (1 μM, 5 min contact), followed by washing, and this process was repeated at least once more.

### Inhibition of cholinergic activity in human colon

Human ascending and descending colon was obtained from patients undergoing surgery for bowel cancer without obstruction or inflammatory bowel disease, in accordance with human ethics regulations and protocols approved by the East London Research Ethics Committee 1 (NREC 09/H0704/2 and 10/H0703/71); written informed consent was obtained from all patients. The tissues were removed at least 10 cm from the tumour and were macroscopically normal, approaching a definition of ‘normal’^21^. The specimens were transferred to the research laboratory within 2 h after resection in Krebs solution equilibrated with 5% CO_2_ and 95% O_2_. Immediately on arrival, the mucosa was removed by blunt dissection and discarded. Strips (~5 mm wide, 12 mm long) were cut parallel to the circular muscle fibres from the inter-taenia region of each colon and were used immediately or after overnight storage at 4 °C in fresh, oxygenated Krebs solution.

The method used to evoke neuromuscular contractions and relaxations of human isolated gastrointestinal tissues by EFS has been described^70^. In brief, strips (4–16 from each patient) were mounted in 10 ml tissue baths containing Krebs solution at 37°, gassed with 5% CO_2_ in O_2_. Changes in muscle tension were recorded in a similar manner to the mouse experiments except that the tissues were suspended under 2 g tension and EFS applied using the parameters of 50 V (c. 200 mA), 0.5 ms bipolar pulse duration, 5 Hz, given for 10 s, every 1 min with the bathing solution changed every 15 min until consistency of responses to EFS was achieved; this has previously been shown to evoke clear, apparently submaximal responses which represent each of the relaxation and contraction phenotypes observed throughout a range of frequencies studied^44^,^70^. Single concentrations of drugs were applied to each muscle strip and their effects determined by measuring at least three EFS-induced responses at a given time-point; changes were expressed as a percentage change from the mean pre-treatment EFS-induced responses. Receptor antagonists and inhibitors were applied for 30 min prior to agonist application. Changes in baseline tension were also expressed as a % of the pre-treatment EFS-induced contraction. The effects of treatments on carbachol-induced contractions were performed after first obtaining consistent responses to 1 μM carbachol (the concentration causing ~50% of maximum contraction to carbachol; 5 min contact, repeated at 15 min intervals) before addition of the drug 15 min before the last application of carbachol.

### Statistical analysis of studies using native tissues

Data are expressed as means ± SD (cell-based assays), medians and ranges (patient characteristics) or as the mean ± standard error of the mean (native tissue experiments) with n values indicating the number of patients or animals. EC_50_ and E_max_ values were obtained from 3 parameter agonist-response curves plotted using GraphPad Prism versions 5.0–6.07; E_max_ values are reported as mean ± standard error. Unpaired Student’s t tests or two way ANOVA models followed by Bonferroni’s multiple comparison tests were used to analyse independent or inter-dependent effects of drugs. The differences between the best fit parameters obtained from the curves were compared using extra sum of squares F tests. P < 0.05 was considered to represent statistical significance.

### Drugs used in native tissue studies

These were freshly prepared prior to use. Carbachol, atropine, L-NAME (N_ω_-nitro-L-arginine methyl ester hydrochloride), propranolol, phentolamine (each from Sigma, UK) and tetrodotoxin (Tocris, UK) were each dissolved in distilled water (dH_2_O). Asimadoline (generous gift from Tioga Inc, USA), ICI204448 and nor-binaltorphimine (both Tocris, UK) were dissolved to 10 mM in DMSO.

### Opioid receptor gene, kappa (OPRK) alignment and tertiary structural analysis

ClustalX was used to align the human, rat and mouse OPRK sequences (uniprot accessions P41145, P34975 and P33534 respectively). Alignments were visualised and annotated for important structural residues using Jalview ( www.jalview.org). A homology modelled structure of human OPRK1 was obtained from the PDB database ( http://www.ebi.ac.uk/pdbe/; PDB id 2a0d). C-terminal rodent residue substitutions were modelled using Swiss PDB viewer ( http://spdbv.vital-it.ch/).

## Additional Information

**How to cite this article**: Broad, J. *et al.* Human native kappa opioid receptor functions not predicted by recombinant receptors: Implications for drug design. *Sci. Rep.* **6**, 30797; doi: 10.1038/srep30797 (2016).

## Supplementary Material

Supplementary Information

## Figures and Tables

**Table 1:** The ability of asimadoline and ICI204448 to recruit G proteins (BRET) and internalise (TR-FRET) human and mouse κ receptors expressed in HEK293 cells.

	Human		Mouse	
	Asimadoline	ICI204448	Asimadoline	ICI204448
BRET				
*p*EC_50_	9.2 ± 0.1	9.6 ± 0.1	8.9 ± 0.1	9.5 ± 0.1
E_max_ (%)	98 ± 5	100 ± 3	99 ± 4	90 ± 4
TR-FRET				
*p*EC_50_	7.4 ± 0.1	7.7 ± 0.1	7.0 ± 0.1	7.5 ± 0.1
E_max_ (%)	97 ± 2	98 ± 2	96 ± 3	92 ± 4

There were no statistically significant differences (P > 0.1) between each measurement when each compound was compared for both the human and mouse receptors (n = 3 replicates).

**Table 2:** Quantification of potential bias in the abilities of asimadoline and ICI204448 to recruit the Gi protein and internalise human and mouse κ receptors expressed in HEK293 cells.

**Gi**					ß-arrestin		
**Agonist**	**Log(max/EC**_**50**_)	**Δlog(max/EC**_**50**_)	**ΔΔLog(max/EC**_**50**_)	**BIAS**	**Δlog(max/EC**_**50**_)	**Log(max/EC**_**50**_)	**Agonist**
Mouse							
ICI204448	9.301					7.301	ICI204448
Asimadoline	9.097	0.204	−0.187	0.65	0.391	6.910	Asimadoline
Human							
ICI204448	9.523					7.481	ICI204448
Asimadoline	9.523	0.000	−0.222	0.60	0.222	7.260	Asimadoline

Mathematical modelling generated log(t/KA) values, a transduction coefficient representing the affinity and efficacy of asimadoline and ICI204448 for either G protein activation or arrestin mobilization, which showed only minimal evidence of biased activity.

**Table 3:** Inhibition by asimadoline and ICI204448 of cholinergically-mediated contractions in mouse isolated colon.

Drug (n)	Asimadoline (3–4)	ICI204448 (3)	Asimadoline + 300 nM nor BNI (3–4)	ICI204448 + 300 nM nor BNI (3–4)
*p*EC_50_	8.1 ± 0.5	8.3 ± 0.5	6.8 ± 0.6	7.0 ± 0.8
E_max_ (%)	52 ± 7	57 ± 7	51 ± 9	44 ± 11

*p*EC_50_ and E_max_ values are given in the absence and presence of nor-binaltorphamine (nor BNI), for which n-values represent numbers of mice or patients studied. Overall, there was no statistically significant difference between the concentration-response curves for asimadoline and ICI204448 obtained in the absence of BNI (P = 0.51).

**Table 4:** Experiments with human colon.

Region of colon	N (strips)	Age (years)	Gender (M : F)	Response to EFS		
				Strips contracting during EFS	Strips relaxing during EFS	Strips with after-contractions
Ascending	32 (280)	69 (42–89)	1 : 3	189	91	164
Descending	43 (462)	63 (35–90)	1 : 0.9	351	111	424

1. Patient demographics and summary of responses to electrical field stimulation (EFS; 5 Hz) for the specimens used in this study. N-values represent numbers of patients.

**Table 5:** Experiments with human colon.

**Ascending Colon**						
	**Treatment (n) concentration**					
	**Asimadoline (4**–**5) 100 pM–10 μM**	**ICI204448 (4) 100 pM–1 μM**	**L-NAME + asimadoline (4) 100 pM–10 μM**	**L-NAME + ICI204448 (4) 100 pM–1 μM**		
*p*EC_50_	7.3 ± 0.4	8.9 ± 0.6	6.4 ± 0.5	8.8 ± 0.3		
E_max_ (%)	108 ± 14	90 ± 15	40 ± 8	52 ± 4		
**Descending Colon**						
	**Treatment (n) concentration**					
	**Asimadoline (4–5) 100 pM–10 μM**	**ICI204448 (4) 100 pM–1 μM**	**L-NAME + asimadoline (4–6) 100 pM–10 μM**	**L-NAME + ICI204448 (4–6) 100 pM–1 μM**		
*p*EC_50_	7.1 ± 0.5	8.0 ± 0.4	6.0 ± 0.3	8.0 ± 0.4		
E_max_ (%)	65 ± 11	108 ± 16	56 ± 11	57 ± 7		

2. Inhibition by asimadoline and ICI204448 of cholinergically-mediated contractions in human ascending and descending colon. pEC_50_ and E_max_ values are given in the absence and presence of L-NAME (300 µM), for which n-values represent numbers of patients studied. Overall, there was a statistically-significant difference in the concentration-response curves for asimadoline and ICI204448 in the absence of L-NAME (ascending P = 0.023, descending P = 0.003) and in the presence of L-NAME (P < 0.0001 for both regions).
