# Supplementary material for: Human native kappa opioid receptor functions not predicted by recombinant receptors: Implications for drug design
Source: Sci Rep. 2016 Aug 5;6:30797. doi: 10.1038/srep30797 (PMC4974614; doi:10.1038/srep30797)

Human native kappa opioid receptor functions not predicted by recombinant receptors: Implications for drug design

John Broad, Damien Maurel, Victor W.S. Kung, Gareth A. Hicks, Michael Schemann, Michael R. Barnes, Terrence P. Kenakin, Sébastien Granier, Gareth J. Sanger

Supplementary Figure 1. A) Homology modelled structure of human OPRK1 (PDB id 2a0d), B) Highlighted human residues in the OPRK1 C-terminal region, C) Highlighted rodent residue substitutions in the OPRK1 C-terminal region

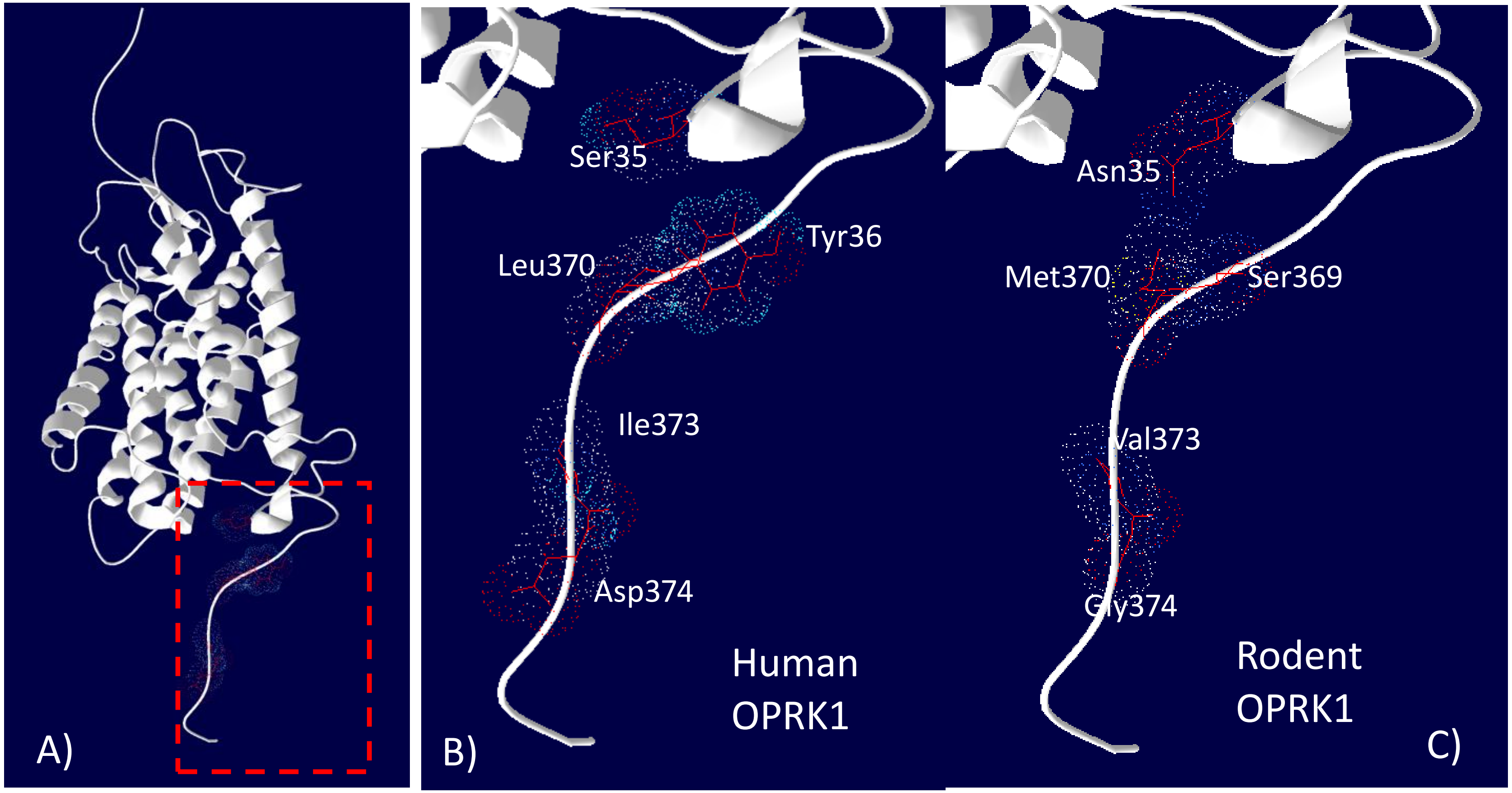

Human native kappa opioid receptor functions not predicted by recombinant receptors: Implications for drug design

John Broad, Damien Maurel, Victor W.S. Kung, Gareth A. Hicks, Michael Schemann, Michael R. Barnes, Terrence P. Kenakin, Sébastien Granier, Gareth J. Sanger

Supplementary Figure 2. Pilot study to examine the abilities of single concentrations of different κ receptor agonists to inhibit cholinergically-mediated contractions of human descending colon evoked by electrical field stimulation. All EC_{50}s are for the human receptor and except where stated are taken from White, K.L. *et al*. (Identification of novel functionally selective κ-opioid receptor scaffolds. Mol. Pharmacol. 85, 83-90, 2014); the remainder (*) are data from the experiments documented in the body of the accompanying paper, measuring ability to recruit G Proteins (BRET) and internalise (TR-FRET) the receptor. Similarly, the data for ICI204448 and asimadoline, obtained using human colon in the presence of L-NAME 300 µM, are from the experiments found in the body of the accompanying paper. For the new experiments with human colon, dynorphin A 1-13, (-) U50488 and U62066 (each from Sigma, UK) were studied using tissue from two patients, again in the presence of L-NAME 300 µM: (1) Rectum, female, 33, cancer; (2) descending colon, male, 51, cancer. All strips displayed contractions during EFS (inhibited by κ agonism) and after-contractions (unaffected by each ligand).

Ligand	Recombinant human receptor		Human intestine			
	G protein pEC_{50}	Arrestin pEC_{50}	Concentration	N	% inhibition of contractions evoked during EFS	Time to max effect (minutes)
Dynorphin 1-13	8.68 ± 0.07	7.01 ± 0.07	1 µM	2	-30, -35	17, 11
(-) U50488	9.06 ± 0.07	9.09 ± 0.09	1 µM	2	-44, -74	31, 27
U62066	9.00 ± 0.05	8.21 ± 0.10	1 µM	2	-39, -50	24, 24
ICI204448	8.38 ± 0.09<br>9.6 ± 0.1*	8.48 ± 0.06<br>7.7 ± 0.1*	1 µM	6	-57 ± 10	13 ± 3
Asimadoline	9.2 ± 0.1*	7.4 ± 0.1*	1 µM	6	-29 ± 4	40 ± 4

Human native kappa opioid receptor functions not predicted by recombinant receptors: Implications for drug design

John Broad, Damien Maurel, Victor W.S. Kung, Gareth A. Hicks, Michael Schemann, Michael R. Barnes, Terrence P. Kenakin, Sébastien Granier, Gareth J. Sanger

Supplementary Figure 3. Detection of SNAP-kappa-opioid receptor at the surface of HEK293 cells. As BG-Tb is not cell permeant, only receptors expressed at the cell surface and displaying the SNAP-tag outside the cell were labelled. Data are means ± SD of a triplicate.

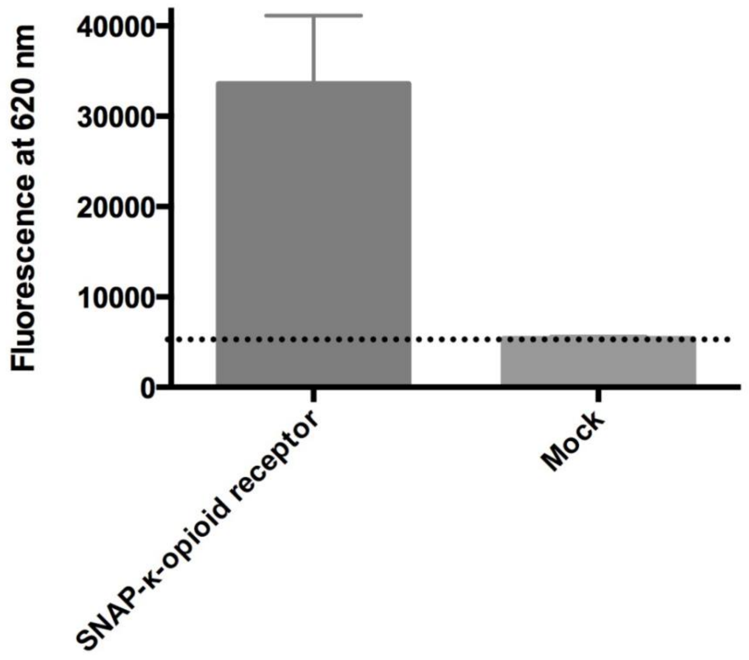

Human native kappa opioid receptor functions not predicted by recombinant receptors: Implications for drug design

John Broad, Damien Maurel, Victor W.S. Kung, Gareth A. Hicks, Michael Schemann, Michael R. Barnes, Terrence P. Kenakin, Sébastien Granier, Gareth J. Sanger

Supplementary Figure 4. BRET was measured in HEK293 cells co-expressing Gαi1-Rluc8, β2 and Venus-γ2 (no receptor). Cells were stimulated with increasing concentrations of ICI204448 and asimadoline. Data are means ± SD of a representative experiment.

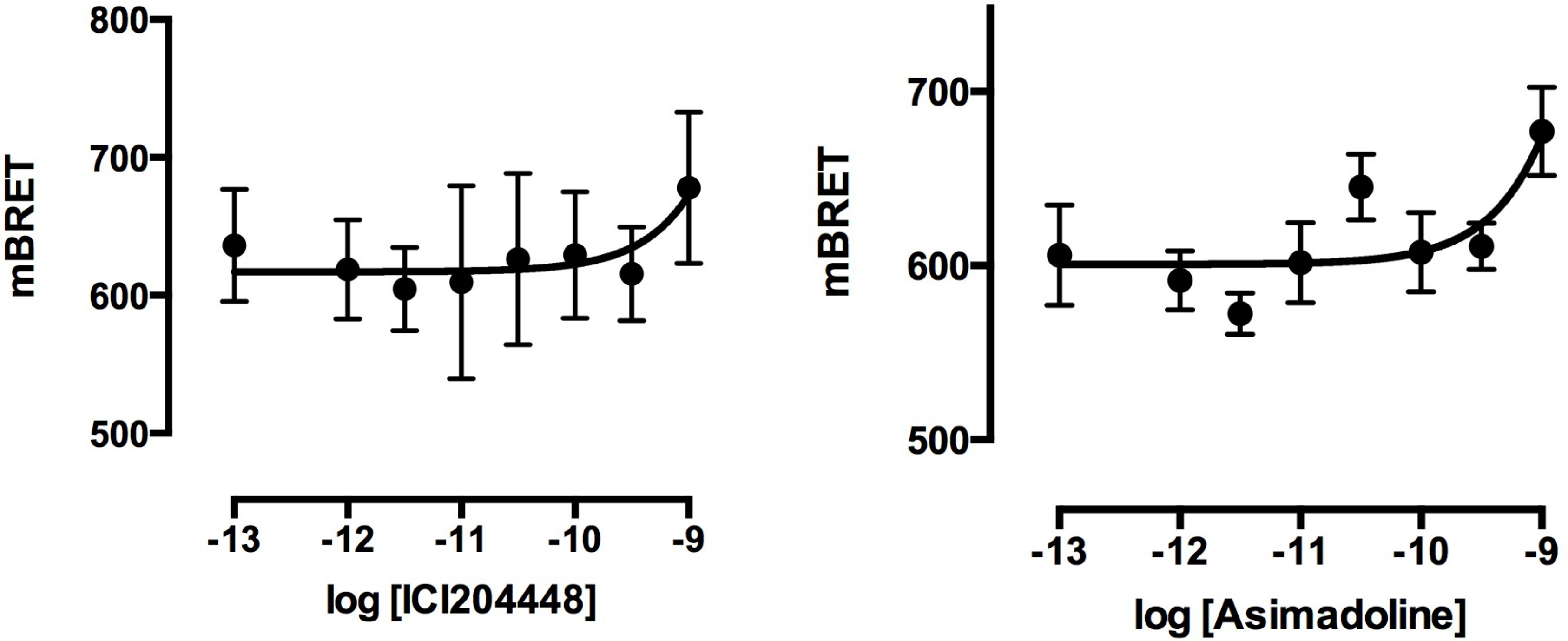

Human native kappa opioid receptor functions not predicted by recombinant receptors: Implications for drug design

John Broad, Damien Maurel, Victor W.S. Kung, Gareth A. Hicks, Michael Schemann, Michael R. Barnes, Terrence P. Kenakin, Sébastien Granier, Gareth J. Sanger

Supplementary Figure 5. (a) Dose response of DALDA on MOR (Gi-coupled receptor) and (b) AVP on V2 (Gs-coupled receptor). DALDA EC50 = 4.10-11M. Data are means ± SD of a representative experiment.

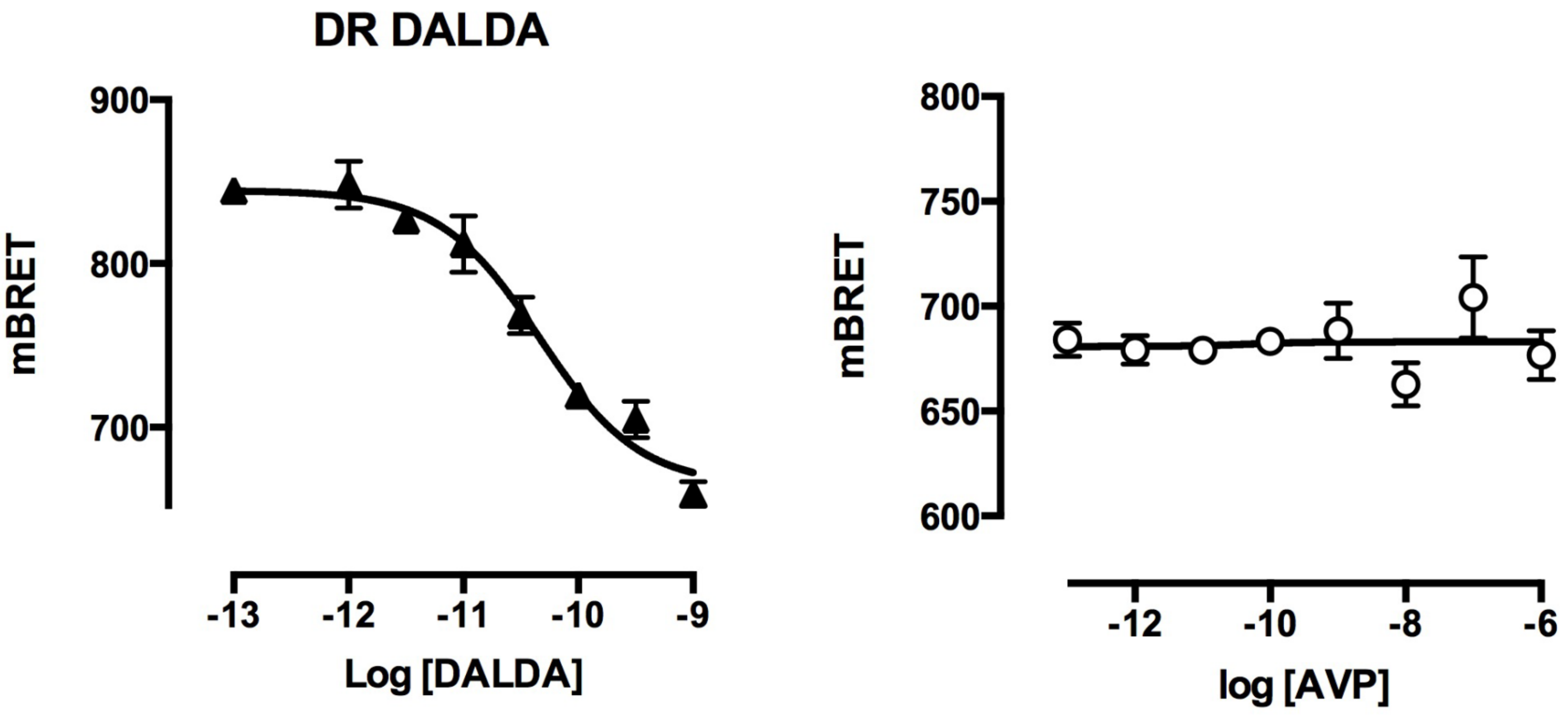

Human native kappa opioid receptor functions not predicted by recombinant receptors: Implications for drug design

John Broad, Damien Maurel, Victor W.S. Kung, Gareth A. Hicks, Michael Schemann, Michael R. Barnes, Terrence P. Kenakin, Sébastien Granier, Gareth J. Sanger

Supplementary Figure 6. a) Time course of V2 internalization with 1μM AVP. b) Dose response of AVP on V2 at T = 40 min. AVP EC50 = 1.10-9M. Data are means ± SD of a representative experiment.

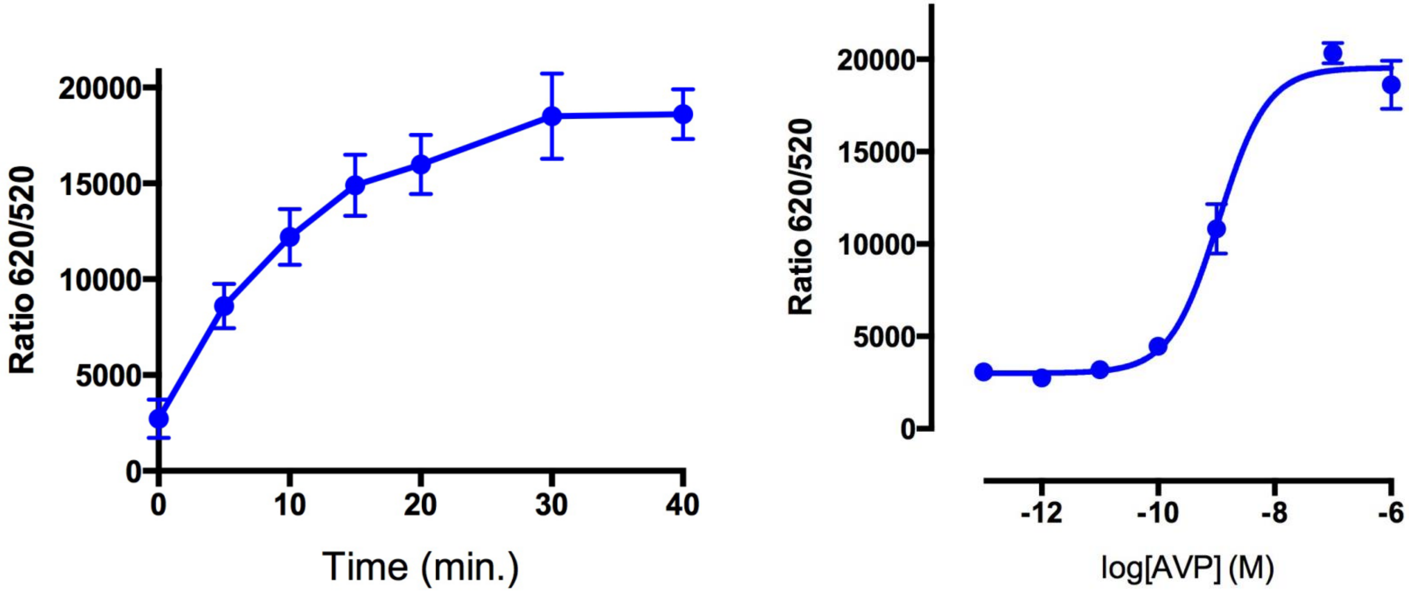

Supplement: Supplementary Information [file srep30797-s1.pdf]
